# Supplementary material for: Association between Ready-to-Eat Cereal Consumption and Nutrient Intake, Nutritional Adequacy, and Diet Quality in Adults in the National Health and Nutrition Examination Survey 2015–2016
Source: Nutrients. 2019 Dec 4;11(12):2952. doi: 10.3390/nu11122952 (PMC6950451; doi:10.3390/nu11122952)
Supplement: Supplementary file 1 [file nutrients-11-02952-s001.pdf]

Supplemental Table S1. Number and percentages of ready-to-eat (RTE) cereal eaters and non-eaters in different National Health and Nutrition Examination Survey cycles.

	18-54 years		55 years or older		18 years or older	
Years	RTE cereal eaters	RTE cereal non-eaters	RTE cereal eaters	RTE cereal non-eaters	RTE cereal eaters	RTE cereal non-eaters
2003-2004	460 (17%)	2370 (83%)	456 (27%)	1418 (73%)	916 (20%)	3788 (80%)
2005-2006	531 (19%)	2502 (81%)	446 (29%)	1199 (71%)	977 (22%)	3701 (78%)
2007-2008	564 (18%)	2724 (82%)	588 (29%)	1727 (71%)	1152 (22%)	4451 (78%)
2009-2010	693 (21%)	2962 (79%)	571 (28%)	1728 (72%)	1264 (24%)	4690 (76%)
2011-2012	518 (19%)	2628 (81%)	431 (29%)	1429 (71%)	949 (22%)	4057 (78%)
2013-2014	541 (18%)	2738 (82%)	415 (24%)	1562 (76%)	956 (20%)	4300 (80%)
2015-2016	449 (16%)	2650 (84%)	382 (23%)	1682 (77%)	831 (19%)	4332 (81%)
2003-2016	3756 (18%)	18574 (82%)	3289 (27%)	10745 (73%)	7045 (21%)	29319 (79%)

Day-1 24-h dietary recall data were used.

Supplemental Table S2. Unadjusted energy intake and nutrient intake in ready-to-eat (RTE) cereal eaters and non-eaters in US adults, National Health and Nutrition Examination Survey 2015-2016.

	18-54 years			≥55 years			≥18 years		
	RTE cereal Eaters	RTE cereal non-eaters	*P*	RTE cereal Eaters	RTE cereal non-eaters	*P*	RTE cereal Eaters	RTE cereal non-eaters	*P*
Energy (kcal)	2250.1±46.6	2195.5±27.2	0.23	2002.1±56.7	1914.1±30.3	0.17	2135.1±35.6	2095.7±22.7	0.29
Total carbohydrate (g)	284.5±9.2	248.3±2.9	0.001	256.7±6.8	218.1±3.4	**0.0003**	271.6±6.4	237.6±2.3	**<0.0001**
Total sugars (g)	128.2±5.2	104.7±2.2	**0.0005**	114.6±5.0	96.2±2.4	0.009	121.9±3.7	101.7±1.7	**<0.0001**
Added sugar (tsp. eq.)	18.3±1.2	16.9±0.5	0.23	14.4±0.9	15.0±0.6	0.62	16.5±0.8	16.3±0.4	0.75
Dietary fiber (g)	21.0±0.8	16.5±0.5	**<0.0001**	21.5±0.8	15.6±0.4	**<0.0001**	21.2±0.5	16.2±0.3	**<0.0001**
Total fat (g)	83.5±2.0	87.9±1.5	0.01	76.5±2.6	79.4±1.7	0.31	80.3±1.3	84.9±1.3	0.002
Saturated fat (g)	28.0±1.0	28.6±0.5	0.50	25.9±1.1	25.8±0.6	0.95	27.0±0.8	27.6±0.5	0.39
Protein (g)	85.4±2.4	87.1±1.9	0.41	76.9±3.2	74.2±1.5	0.48	81.4±2.3	82.5±1.3	0.68
Calcium (mg)	1233.9±41.2	948.2±22.2	**<0.0001**	1091.3±33.7	802.0±26.5	**<0.0001**	1167.8±26.0	896.3±18.9	**<0.0001**
Iron (mg)	21.3±0.7	12.9±0.2	**<0.0001**	20.7±0.5	11.4±0.2	**<0.0001**	21.0±0.5	12.3±0.1	**<0.0001**
Magnesium (mg)	348.1±9.9	306.7±6.9	0.0007	331.1±10.6	277.2±6.4	**0.0006**	340.2±6.3	296.3±5.9	**<0.0001**
Potassium (mg)	2905.5±96.8	2585.4±48.4	0.002	2979.4±104.6	2473.0±41.9	**0.0001**	2939.8±76.1	2545.6±36.9	**<0.0001**
Sodium (mg)	3508.6±118.7	3779.8±56.1	0.03	3164.3±112.7	3223.3±76.6	0.69	3348.9±96.1	3582.4±49.6	0.06
Zinc (mg)	14.6±0.5	11.0±0.2	**<0.0001**	13.2±0.5	9.7±0.2	**<0.0001**	13.9±0.4	10.5±0.2	**<0.0001**
Vitamin A, RAE (mcg)	1020.1±80.1	556.4±15.6	**<0.0001**	844.7±62.3	541.9±17.0	**0.0007**	938.7±42.9	551.2±12.8	**<0.0001**
Thiamin (mg)	2.1±0.1	1.5±0.0	**<0.0001**	1.9±0.1	1.4±0.0	**<0.0001**	2.0±0.0	1.5±0.0	**<0.0001**
Riboflavin (mg)	2.9±0.1	2.1±0.0	**<0.0001**	2.6±0.1	1.9±0.1	**<0.0001**	2.8±0.1	2.0±0.0	**<0.0001**
Niacin (mg)	31.7±1.0	27.4±0.5	**0.0004**	27.3±0.8	21.4±0.7	**<0.0001**	29.7±0.7	25.3±0.5	**<0.0001**
Vitamin B_6 (mg)	3.1±0.1	2.2±0.1	**<0.0001**	2.6±0.1	1.6±0.1	**<0.0001**	2.9±0.1	2.0±0.0	**<0.0001**
Folate, DFE (mcg)	915.9±30.4	458.5±12.1	**<0.0001**	773.8±24.2	388.9±9.0	**<0.0001**	850.0±21.4	433.8±7.9	**<0.0001**
Vitamin B_{12} (mcg)	7.9±0.3	4.6±0.1	**<0.0001**	7.2±0.9	3.8±0.2	0.003	7.6±0.4	4.3±0.1	**<0.0001**
Vitamin C (mg)	90.0±6.7	78.1±3.6	0.08	98.2±7.3	71.2±3.3	0.002	93.8±4.8	75.6±2.5	**0.0007**
Vitamin D (mcg)	8.0±0.6	4.0±0.2	**<0.0001**	6.9±0.5	3.9±0.2	**<0.0001**	7.5±0.4	4.0±0.1	**<0.0001**
Vitamin E as alpha-tocopherol (mg)	11.2±1.0	9.3±0.3	0.06	9.2±0.4	8.2±0.3	0.06	10.3±0.6	8.9±0.2	0.02

Day-1 24-h dietary recall data were used. P value <0.05/48=0.001 was considered to be statistically significant and shown in bold.

Supplemental Table S3. Adjusted energy intake and nutrient intake in ready-to-eat (RTE) cereal eaters and non-eaters in US adults aged 18 years or older, National Health and Nutrition Examination Survey 2015-2016.

	RTE cereal Eaters	RTE cereal non-eaters	*P*
Energy (kcal)	2143.5±33.4	2054.6±26.7	0.02
Total carbohydrate (g)	276.0±3.3	243.9±1.6	**<0.0001**
Total sugars (g)	121.8±2.9	102.0±2.2	**<0.0001**
Added sugar (tsp. eq.)	16.4±0.7	15.8±0.6	0.22
Dietary fiber (g)	21.1±0.7	16.8±0.4	**<0.0001**
Total fat (g)	70.4±1.6	78.4±0.6	**<0.0001**
Saturated fat (g)	23.8±0.6	25.5±0.3	0.01
Protein (g)	79.6±2.1	82.1±0.8	0.15
Calcium (mg)	1143.2±24.3	895.6±12.2	**<0.0001**
Iron (mg)	20.7±0.5	12.3±0.1	**<0.0001**
Magnesium (mg)	387.4±6.7	352.9±4.3	**0.0006**
Potassium (mg)	2979.9±62.3	2680.8±25.0	**0.0001**
Sodium (mg)	3201.7±59.1	3494.9±35.6	**0.0006**
Zinc (mg)	13.5±0.3	10.3±0.1	**<0.0001**
Vitamin A, RAE (mcg)	900.1±53.6	538.8±19.8	**<0.0001**
Thiamin (mg)	1.9±0.1	1.4±0.0	**<0.0001**
Riboflavin (mg)	2.9±0.1	2.2±0.0	**<0.0001**
Niacin (mg)	34.5±0.6	29.9±0.6	**<0.0001**
Vitamin B_6 (mg)	3.1±0.1	2.2±0.0	**<0.0001**
Folate, DFE (mcg)	899.7±23.0	486.3±5.9	**<0.0001**
Vitamin B_{12} (mcg)	7.7±0.4	4.5±0.2	**<0.0001**
Vitamin C (mg)	104.5±4.4	89.4±2.5	0.004
Vitamin D (mcg)	7.7±0.4	4.3±0.2	**<0.0001**
Vitamin E as alpha-tocopherol (mg)	9.4±0.6	8.5±0.2	0.13

Day-1 24-h dietary recall data were used. Covariates included age, gender, race/ethnicity, ratio of family income to poverty, education level, and smoking status. Energy intake was also included as another covariate in the analysis of nutrients. P value <0.05/24=0.002 was considered to be statistically significant and shown in bold.

Supplemental Table S4. Adjusted energy intake and nutrient intake by frequency of Ready-to-eat (RTE) cereal consumption in US adults aged 18 years or older, National Health and Nutrition Examination Survey 2015-2016.

	Non-eaters (n=3212)	RTE cereal consumption in one day (n=744)	RTE cereal consumption in two days (n=362)	*P*
Energy (kcal)	1994.1±19.4^{a}	2145.8±46.3^{a}	2061.3±39.7^{a}	0.01
Total carbohydrate (g)	229.8±1.8^{a}	244.6±3.4^{b}	260.1±3.1^{c}	**<0.0001**
Total sugars (g)	95.1±1.3^{a}	98.5±3.1^{a}	113.6±2.9^{b}	**0.0001**
Added sugar (tsp. eq.)	14.5±0.3^{a}	13.4±0.7^{a}	14.2±0.5^{a}	0.39
Dietary fiber (g)	16.5±0.2^{a}	19.1±0.6^{b}	21.3±0.3^{c}	**<0.0001**
Total fat (g)	79.1±0.5^{a}	75.3±1.1^{a,b}	70.5±1.1^{b}	**<0.0001**
Saturated fat (g)	25.1±0.2^{a}	24.1±0.6^{a,b}	23.1±0.3^{b}	**0.0005**
Protein (g)	80.4±0.7^{a}	77.2±1.2^{a}	81.5±1.8^{a}	0.06
Calcium (mg)	831.4±7.1^{a}	969.7±21.1^{b}	1120.9±29.2^{c}	**<0.0001**
Iron (mg)	12.0±0.1^{a}	16.7±0.4^{b}	21.7±0.4^{c}	**<0.0001**
Magnesium (mg)	287.1±3.8^{a}	311.5±9.0^{a,b}	329.9±7.6^{b}	**0.0001**
Potassium (mg)	2489.3±20.3^{a}	2590.1±51.6^{a}	2841.6±38.9^{b}	**<0.0001**
Sodium (mg)	3432.6±25.3^{a}	3231.8±57.9^{a}	3157.3±83.7^{a}	0.003
Zinc (mg)	10.2±0.1^{a}	11.1±0.2^{b}	14.2±0.4^{c}	**<0.0001**
Vitamin A, RAE (mcg)	539.5±14.1^{a}	674.5±34.2^{b}	823.4±23.4^{c}	**<0.0001**

Thiamin (mg)	1.4±0.0^{a}	1.6±0.0^{b}	2.0±0.1^{c}	**<0.0001**
Riboflavin (mg)	1.9±0.1^{a}	2.2±0.1^{b}	2.6±0.1^{c}	**<0.0001**
Niacin (mg)	24.1±0.4^{a}	25.8±0.6^{a}	30.1±0.8^{b}	**<0.0001**
Vitamin B_6 (mg)	1.9±0.0^{a}	2.2±0.1^{b}	2.8±0.1^{c}	**<0.0001**
Folate, DFE (mcg)	420.0±6.1^{a}	629.6±14.9^{b}	872.9±23.4^{c}	**<0.0001**
Vitamin B_{12} (mcg)	4.2±0.2^{a}	5.2±0.2^{b}	7.7±0.4^{c}	**<0.0001**
Vitamin C (mg)	80.9±2.5^{a}	83.8±5.7^{a}	89.8±3.6^{a}	0.11
Vitamin D (mcg)	4.0±0.1^{a}	5.6±0.2^{b}	7.9±0.4^{c}	**<0.0001**
Vitamin E as alpha-tocopherol (mg)	8.3±0.2^{a}	9.0±0.6^{a}	9.1±0.6^{a}	0.22

Two-day 24-h dietary recall data were used. Covariates included age, gender, race/ethnicity, ratio of family income to poverty. Energy intake was also included as another covariate in the analysis of nutrients. P value <0.05/(24*3)=0.0007 was considered to be statistically significant and shown in bold. Different letters in the same row indicate significant differences between groups.

Supplemental Table S5. Contribution of ready-to-eat cereal and co-consumed milk to daily intake of energy and nutrients in ready-to-eat cereal and milk eaters, National Health and Nutrition Examination Survey 2015-2016.

	18-54 years	55 years or older	18 years or older
Energy	14%	13%	14%
Carbohydrate	20%	19%	20%
Total sugar	20%	17%	19%
Added sugar	18%	15%	17%
Dietary fiber	21%	24%	22%
Total fat	8%	7%	7%
Saturated fat	10%	8%	9%
Protein	13%	12%	13%
Calcium	25%	24%	25%
Iron	49%	51%	50%
Magnesium	19%	19%	19%
Potassium	16%	14%	15%
Sodium	9%	9%	9%
Zinc	36%	34%	35%
Vitamin A	38%	36%	37%
Thiamin	37%	34%	36%
Riboflavin	37%	32%	35%
Niacin	30%	30%	30%
Vitamin B6	44%	41%	43%
Folate	56%	54%	55%
Vitamin B12	53%	47%	50%
Vitamin C	11%	9%	10%
Vitamin D	51%	47%	50%
Vitamin E	21%	21%	21%

Day-1 24-h dietary recall data were used.

Supplemental Table S6. General health status and selected health conditions in ready-to-eat (RTE) cereal eaters and non-eaters in US adults aged 18 years or older, National Health and Nutrition Examination Survey 2015-2016.

	RTE cereal eaters[1]	RTE cereal non-eaters	*P*[2]
General Health Status[3]			**0.037**
Good or excellent	628 (83%)	3035 (78%)	
Fair	133 (12%)	942 (16%)	
Poor	27 (2%)	146 (2%)	
Missing	43 (3%)	209 (4%)	
Overweight/Obese[4]			**0.030**

Yes	567 (68%)	3107 (72%)	
No	264 (32%)	1225 (28%)	
Diabetes[5]			0.575
Yes	120 (13%)	627 (11%)	
No	691 (85%)	3611 (87%)	
Borderline	20 (2%)	94 (2%)	
Hypertension[6]			0.213
Yes	297 (34%)	1551 (31%)	
No	533 (66%)	2777 (69%)	
Don't know	1 (0%)	4 (0%)	

[1]RTE cereal eaters were defined if they reported RTE cereal consumption in day 1 dietary recall data. [2]P value from survey Chi-square test. [3]Based on responses to the question "would you say your health in general is…" in the Hospital Utilization & Access to Care Questionnaire. [4]Overweight/Obese is defined as body mass index ≥ 25 kg/m^2. [5]Based on responses to the question "have you ever been told by a doctor or other health professional that you have diabetes or sugar diabetes" in the Diabetes Questionnaire. [6] Based on responses to the question "have you ever been told by a doctor or other health professional that you have hypertension or high blood pressure" in the Blood Pressure & Cholesterol Questionnaire.
